# Strength and Ductility Enhancement in Coarse-Aggregate UHPC via Fiber Hybridization: Micro-Mechanistic Insights and Artificial Neural Network Prediction

**DOI:** 10.3390/ma19010157

**Published:** 2026-01-02

**Authors:** Jiyang Wang, Yalong Wang, Shubin Wang, Yijian Zhan, Yu Peng, Zhihua Hu, Bo Zhang

**Affiliations:** 1 Institute of Advanced Engineering Structures, Zhejiang University, Hangzhou 310058, China; 22312290@zju.edu.cn (Y.W.); zjupengyu@zju.edu.cn (Y.P.); zhihuah@zju.edu.cn (Z.H.); 2 Center for Balance Architecture, Zhejiang University, Hangzhou 310007, China; wangshubin1967@126.com; 3 The Architectural Design & Research Institute of Zhejiang University Co., Ltd., Hangzhou 310007, China; 4 Central Research Institute, Shanghai Construction Group Co., Ltd., Shanghai 200080, China; 5 Ocean Research Center of Zhoushan, Zhejiang University, Zhoushan 316021, China; zb911@zju.edu.cn

**Keywords:** ultra-high-performance concrete, coarse aggregate, artificial neural network, ductility, hybrid fiber, micro-mechanism, orthogonal experimental design

## Abstract

Incorporating coarse aggregates into ultra-high-performance concrete (UHPC-CA) can reduce material costs, yet reliably predicting its strength-related behavior and overall performance remains challenging. This study examines UHPC-CA through a two-stage orthogonal experimental program comprising 18 mixtures with coarse aggregate, fly ash, and hybrid fiber reinforcements (steel, polypropylene, and composite fibers). Microstructural characterization using scanning electron microscope (SEM) and X-ray computed tomography (X-CT) was conducted to assess interfacial features and crack evolution and to link these observations to the measured mechanical response. Experimentally, fiber reinforcement markedly enhanced post-cracking performance. Compared with the fiber-free control mixture, the optimal hybrid configuration increased flexural strength from 6.9 to 23.5 MPa and compressive strength from 60.1 to 90.5 MPa. The steel–composite fiber system outperformed the steel–polypropylene system, which is consistent with the tighter composite-fiber interfacial bonding observed by SEM/X-CT and supports the feasibility of partially substituting steel fibers. An artificial neural network (ANN) model trained on 50 mixtures and evaluated on 10 unseen mixtures achieved an R^2^ of 0.9703, an MAE of 1.22 MPa, and an RMSE of 2.11 MPa for compressive strength prediction, enabling sensitivity assessment under multi-factor coupling. Overall, the proposed experiment–characterization–modeling framework provides a data-driven basis for performance-oriented mix design and rapid screening of UHPC-CA.

## 1. Introduction

Concrete remains the most extensively utilized construction material worldwide [1]. To address the inherent brittleness and limited tensile capacity of ordinary concrete, Larrard et al. [2] pioneered the development of ultra-high-performance concrete (UHPC) in 1994. UHPC exhibits exceptional strength, ductility, and durability through the meticulous optimization of particle-size distribution, the incorporation of ultra-fine supplementary cementitious materials (e.g., silica fume and fly ash), the use of a low water-to-binder ratio, the implementation of rigorous curing regimes, and the inclusion of steel fibers. Since its introduction, UHPC has been successfully applied across a broad range of structural engineering contexts [3]. UHPC has been effectively utilized not only for the seismic retrofitting of building structures [4] but also for enhancing their resistance to blast and impact loads [5]. However, its exceptionally high binder content increases the heat of hydration and induces substantial autogenous shrinkage (typically 400–800 με), thereby increasing the risk of early-age cracking [6]. Moreover, the high material cost continues to hinder its large-scale practical adoption.

Introducing coarse aggregate into UHPC—yielding UHPC with coarse aggregate (UHPC-CA)—is widely regarded as a cost–performance compromise. Coarse aggregate reduces both the dosage and unit cost of cementitious binders and substantially lowers autogenous shrinkage (by approximately 40%) [7]. Nevertheless, Li et al. [8] reported a modest reduction in the mechanical properties of UHPC upon adding coarse aggregate. However, incorporating coarse aggregates also introduces structural-theoretical challenges, particularly regarding increased heterogeneity, potential stress concentrations at the ITZ, and the complexity of predicting fracture behavior under such multiphase interactions. Conventional UHPC typically employs a single steel fiber type; however, the incremental benefits of single-fiber reinforcement are limited. Hybrid fiber strategies that combine steel and polymeric fibers can leverage the complementary behaviors of stiff (high-modulus) and compliant (low-modulus) fibers, while partially substituting steel with lower-cost polymer fibers to manage material costs. Current research on fiber hybridization in UHPC follows two main paths. (1) Hybridizing steel fibers of different geometries or lengths: Kim et al. [9] showed that incorporating 1.5 vol.% straight fibers plus 0.5 vol.% hook-ended fibers improved compressive, tensile, and flexural strengths, with the most pronounced gains in toughness; Wei et al. [10] found that 1.5 vol.% long steel fibers combined with 1.0 vol.% short steel fibers produced the highest flexural strengths. Overall, geometry-based or length-based blends can be selected to meet specific performance targets; however, the improvements are generally modest. (2) Combining steel fibers with polymeric fibers: the elastic-modulus contrast enables complementary crack-bridging across different crack widths and strain levels [11], and partial replacement of steel with relatively inexpensive polymer fibers reduces cost. Standard polymeric fibers used in conjunction with steel fibers include polyvinyl alcohol (PVA), polyethylene (PE), and polypropylene (PP). Zhang et al. [12] reported that steel–PVA hybrids slightly reduce compressive strength but effectively enhance flexural strength; similar trends were observed for steel–PE hybrids by Zhou et al. [13]. Currently, the steel–PP system has attracted the most attention, as it not only improves the basic mechanical properties of UHPC [14] but also markedly enhances resistance to explosive spalling at elevated temperatures [15] and improves fatigue performance [16]. Deng et al. [17] and Ravichandran et al. [18] provided a comprehensive review of the impact of diverse fiber types on UHPC performance through matrix interactions, alongside an analysis of implementation challenges and future trends. Collectively, these studies indicate that mixing steel fibers with polymeric fibers typically entails a modest reduction in compressive strength in exchange for notable gains in other performance metrics. While fibers enhance ductility, incorporating fly ash not only reduces cement consumption but also improves matrix workability and density, potentially creating a more favorable bond environment for the hybrid fiber system.

Accurate and repeatable prediction of UHPC mechanical properties is essential for rapidly optimizing hybrid-fiber mix proportions and achieving a performance–cost balance [19]. Recent work increasingly applies machine learning (ML) to UHPC property prediction, with compressive strength—the primary target metric—receiving the most attention [20]. Algorithms such as artificial neural networks (ANN) [21,22], support vector machines (SVM) [23,24], decision trees (DT) [25,26], and genetic algorithms (GA) [27] have shown strong predictive performance across concrete datasets. Despite progress on UHPC with coarse aggregate (UHPC-CA) [28,29], key gaps persist: the optimal coarse aggregate content, polypropylene dosage in steel–PP hybrids, systematic optimization of ultrafine reactive supplementary cementitious materials (e.g., fly ash), and head-to-head comparisons of alternative fiber architectures. For UHPC-CA strength prediction, studies often underreport modeling details (data curation, feature engineering, training protocols) and provide limited evidence of out-of-sample generalization. Moreover, a unified framework that integrates experimentation, characterization, and modeling to validate relationships across scales—whereby mix design influences microstructure and microstructure governs strength under multifactor coupling—is still lacking.

Building on prior work, this study aims to establish a comprehensive experimental–characterization–modeling framework for UHPC-CA. Beyond systematically optimizing the mix design of coarse aggregates and hybrid fibers for flowability and strength, the study seeks to elucidate the microstructural mechanisms governing these properties through ITZ and crack analysis. This experimental foundation supports the development of an ANN model that captures the nonlinear sensitivity of multi-factor couplings and provides a robust, data-driven tool for rapid engineering screening and optimization of UHPC-CA. In this study, the ML component is presented after the experimental and microstructural analyses to ensure that data-driven prediction supports—rather than replaces—the mechanism-based interpretation.

## 2. Materials and Methods

### 2.1. Raw Materials

**Figure 1:**
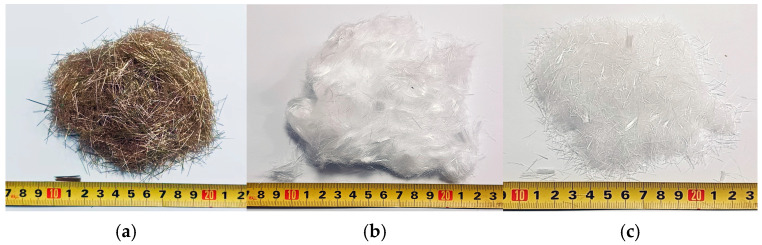
Morphology of different fibers: (**a**) steel fibers; (**b**) polypropylene fiber; (**c**) polyethylene–polypropylene composite fibers.

The raw materials used in this study comprised Portland cement, fly ash, silica fume, sand, coarse aggregate, steel fibers, polypropylene fibers, composite fibers, plasticizers, and water. The cement was P·II 52.5 Portland cement (Tongling Conch Cement Co., Ltd., Tongling, China) with a specific surface area of 372.9 m^2^/kg. The fly ash was Grade I (China Jianbi Power Plant, Zhenjiang, China), and the silica fume was a micro-powder supplied by Eken International Trading Co., Ltd. (Shenzhen, China). The microsilica fume contains 94.0% silica (SiO_2_), 0.4% moisture, and a loss on ignition of 0.5%. The fine aggregate was a calcined medium silica sand with a 40/70 mesh gradation (Tongliao Hongyuan Silica Sand Co., Ltd., Tongliao, China), and the coarse aggregate was crushed stone with a nominal size range of 5–10 mm. Copper-coated steel fibers were sourced from Zhejiang Boren Metal Products Co., Ltd. (Huzhou, China). Polypropylene fibers with a density of 0.91 g/cm^3^ were used as received, and polyethylene–polypropylene composite fibers (Shike TMA12, Ningbo Shike New Materials Technology Co., Ltd., Ningbo, China) served as the composite fiber type, achieving a break elongation of 7.0%. A BASF dry-powder plasticizer was employed, and tap water was used for mixing. The chemical compositions of the raw materials are detailed in Table 1, Table 2 and Table 3. Furthermore, the physical properties and morphologies of the different fiber types are presented in Table 4 and Figure 1, respectively. All materials are standard industrial-grade and comply with the relevant quality specifications, thereby ensuring the reliability of the reported baseline properties.

### 2.2. Orthogonal Experimental Design

To systematically investigate the multifactorial influences on the mechanical properties of UHPC-CA, this study employed an orthogonal experimental design. This methodology enables efficient determination of main effects and interactions among factors by analyzing a representative subset of experimental combinations [30]. The investigation consisted of two sequential L_9_(3^3^) orthogonal arrays. The first array, conducted with a fixed water-binder ratio of 0.33, examined the influence of three factors: coarse aggregate content (10%, 15%, and 20% by mass), steel fiber volume fraction (0%, 1%, and 2%), and polypropylene fiber volume fraction (0%, 0.1%, and 0.2%). Subsequently, the second array was designed to optimize the cementitious material-fiber systems. This phase utilized the optimal coarse aggregate content determined from the first array and a reduced water-binder ratio of 0.27. It investigated the effects of the fly ash-to-cement mass ratio (21%, 33%, and 42%), steel fiber volume fraction (1.0%, 1.25%, and 1.5%), and a composite fiber volume fraction (0.3%, 0.4%, and 0.5%). The fiber dosage ranges (0–2.0% for steel and 0–0.5% for composite fiber) were established to maximize mechanical enhancement while preventing fiber agglomeration and ensuring sufficient workability for the coarse aggregate matrix. The water binder ratios of 0.27 and 0.33 were specifically selected to evaluate the trade-off between matrix densification (strength) and the rheological requirements for suspending coarse aggregates (workability). Although the L_9_(3^3^) orthogonal design focuses on main effects and does not explicitly quantify interaction terms, it was selected to screen for dominant parameters efficiently and to establish a data foundation for subsequent nonlinear ANN modeling. The mix proportions for the benchmark and orthogonal experimental groups are provided in Table 5, Table 6 and Table 7. Table 5 details the benchmark designs, while Table 6 and Table 7 present the orthogonal mix designs alongside their respective test results. In each group, three specimens were prepared.

### 2.3. Specimen Preparation and Curing

The fabrication of all specimens was conducted in strict accordance with the T/CBMF 37-2018 standard [31]. The mixing sequence began with dry mixing the pre-weighed fine and coarse aggregates for approximately 30 s to ensure uniform dispersion. Cement, fly ash, and silica fume were then added and dry-mixed for an additional 1 min. Subsequently, two-thirds of the total water and the entire superplasticizer dosage were gradually introduced while mixing for 3 min, until the mixture reached a fluid consistency. Then add polypropylene or hybrid fibers along with the remaining water, and blend for an additional 3 min. Finally, the steel fibers were slowly incorporated, followed by a final 5 min mixing period to ensure their homogeneous distribution.

The fresh UHPC-CA mixture was cast into two types of oiled steel molds: 70.7 mm × 70.7 mm × 70.7 mm cubes for compressive strength tests and 40 mm × 40 mm × 160 mm prisms for flexural strength tests. During casting, a small vibrating table is used to compact the specimens and eliminate entrapped air. Immediately after, the exposed surfaces were covered with plastic film to prevent moisture loss. The specimens were demolded after 24 h, and subsequently submerged in water for 28 days of standard curing. For each mix design, three specimens of each type were prepared to ensure the reliability of the test results.

### 2.4. Mechanical Property Testing

The mechanical properties of UHPC-CA were evaluated in accordance with GB/T 50081-2019 [32] (which specifies testing protocols consistent with ASTM C1856/C1856M-17 [33] for high-strength cementitious composites). Compressive strength tests were performed on the cubic specimens using a YAW-3000 electro-hydraulic pressure Tester (Jinan Liangong Testing Technology Co., Ltd., Jinan, China) at a constant loading rate of 0.8–1.0 MPa/s. Flexural strength was determined using a three-point bending test on prismatic specimens, conducted on a YYW-300DS testing machine at a loading rate of 0.08–0.10 MPa/s. The flexural strength, *f*_f_, was calculated using Equation (1):

(1) $$f_{f}=\frac{3Fl}{2bh^{2}}$$

where *F* is the fracture load (unit: N), *l* is the support span (unit: mm), and *b* and *h* are the width and height of the specimen’s cross-section (unit: mm), respectively.

### 2.5. Microstructural Characterization

Microstructural characterization was performed using scanning electron microscopy (SEM) and X-ray computed tomography (X-CT). SEM observations were conducted using a field-emission environmental SEM (FEI QUANTA FEG 650, FEI company, Hillsboro, OR, USA). Samples were extracted from fractured specimens, and representative fractured fragments (approximately 5 mm in size) were selected and mounted on SEM stubs using thermal adhesive. The SEM was operated at an accelerating voltage of 10 kV. The natural fracture surfaces were examined directly, without grinding or polishing, to preserve the original fracture morphology and to characterize the interfacial transition zones (ITZs) and the bond quality between the matrix and the coarse aggregates, steel fibers, and polymer fibers, including fiber–matrix debonding and pull-out features.

Furthermore, the internal structure and failure mechanisms were investigated non-destructively using an X-CT system (NIKON XTH 320/225 LC CT, NIKON, Tokyo, Japan). Scans were performed on fractured flexural specimens at a tube voltage of 160 kV and a current of 120 μA, achieving an effective voxel resolution of approximately 31.2 μm. Tomographic reconstruction and subsequent analysis were carried out using VGStudio MAX (version 3.1). Three-dimensional crack propagation patterns were visualized, and the spatial distribution and orientation of steel fibers were quantified by segmenting the fibers from the cementitious matrix based on distinct peaks in the grayscale histogram.

## 3. Results and Discussion

### 3.1. Failure Modes and Mechanisms

**Figure 2:**
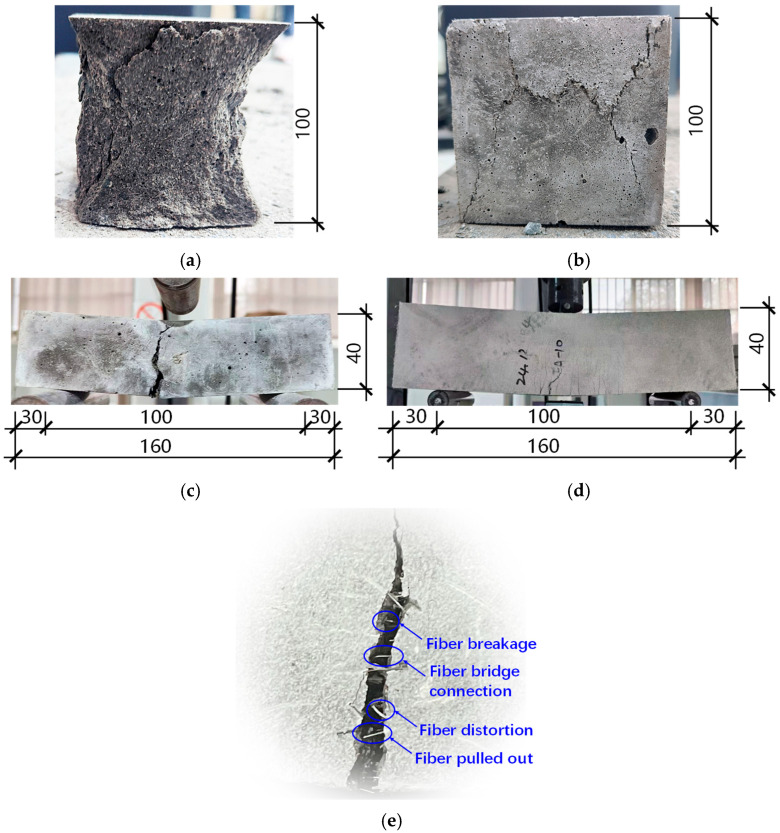
Typical failure modes: (**a**) compressive and (**c**) flexural failure of conventional high-strength concrete; (**b**) compressive and (**d**) flexural failure of UHPC-CA with hybrid fibers; (**e**) different forms of fibers at crack locations. Unit: mm.

Figure 2 contrasts the failure modes of plain and fiber-reinforced UHPC-CA specimens under compressive and flexural loading. Plain concrete exhibited typical brittle failure: upon reaching peak load, rapid crack propagation led to extensive surface spalling in compression (Figure 2a) and catastrophic bifurcation in flexure (Figure 2c). In contrast, fiber-reinforced specimens demonstrated a marked transition from brittle to ductile behavior, characterized by superior post-cracking integrity and sustained load-carrying capacity (Figure 2b,d). This improved performance is attributed to the crack-bridging effect of the fibers, which arrests crack opening and prevents the severe spalling and full-section separation observed in unreinforced specimens.

Microscopic inspection of the fracture surfaces reveals that energy is dissipated primarily through fiber pull-out and rupture (Figure 2e), confirming the activation of toughening mechanisms. Notably, the macroscopic failure patterns were similar for both the steel–polypropylene and steel–composite hybrid systems. This similarity suggests that the high-stiffness steel fibers dominate macro-crack bridging and dictate the ultimate failure mode. Conversely, the lower-modulus polymeric fibers (polypropylene or composite types) primarily control microcrack initiation and coalescence within the matrix. Therefore, while steel fibers govern the macroscopic failure signature, the observed enhancements in ductility and damage tolerance result from the synergistic interaction between the macro-reinforcement provided by steel fibers and the micro-confinement of the polymeric phase.

### 3.2. Range Analysis and Mechanistic Interpretation

#### 3.2.1. Range Analysis Results and Comprehensive Evaluation

**Figure 3:**
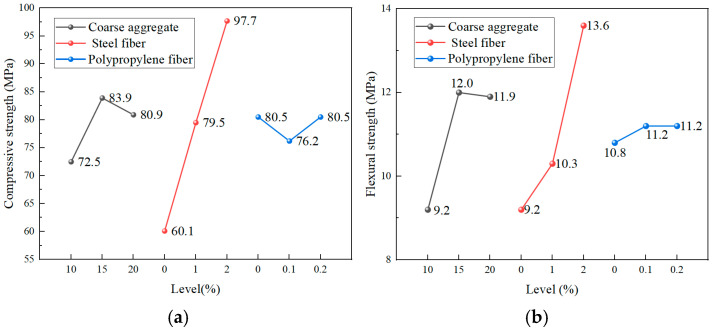
Range analysis of Phase I experiments: (**a**) Compressive strength; (**b**) Flexural strength.

**Figure 4:**
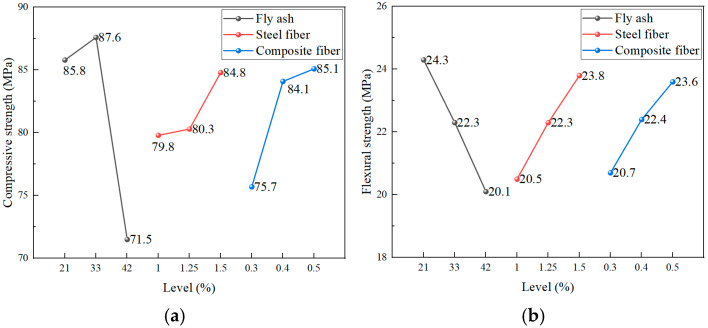
Range analysis of Phase II experiments: (**a**) Compressive strength; (**b**) Flexural strength.

The compressive and flexural strengths obtained from the two-phase experimental program are summarized in Table 6 and Table 7. Subsequently, range analyses were conducted to determine the optimal mix proportions, and the detailed results are presented in Table 8 and Table 9. The corresponding range analysis plots (Figure 3 and Figure 4) illustrate the influence patterns and relative significance of the key mix design parameters governing the mechanical behavior of UHPC-CA.

##### Phase I: Optimization of the Aggregate–Fiber System

Phase I evaluated the influence of coarse aggregate content and hybrid fiber dosage (steel and polypropylene) on the mechanical performance of UHPC-CA. Range analysis (Table 8) indicates that the sensitivity of both compressive and flexural strengths to the investigated factors follows the order: steel fiber > coarse aggregate > polypropylene fiber.

For compressive strength, increasing the coarse aggregate content from 10% to 15% resulted in a 15.7% gain. However, further expanding the content to 20% yielded only an 11.6% return, indicating that 15% is the optimal aggregate dosage. Steel fibers demonstrated a dominant strengthening effect, with 1% and 2% additions enhancing compressive strength by 32.3% and 62.6%, respectively, relative to the control. In contrast, polypropylene fibers had a limited impact; a 0.1% addition reduced strength by 5.3%, whereas 0.2% maintained strength comparable to the control, suggesting 0.2% as the preferable dosage.

Flexural behavior followed a similar trend. Flexural strength peaked at 15% coarse aggregate content, with a 30.4% increase relative to the 10% level. Steel fibers at 2% dosage enhanced flexural strength by 47.8%. Polypropylene fibers made a marginal contribution, with dosages of 0.1% and 0.2% increasing strength by approximately 3.7%. Based on a comprehensive evaluation of the Phase I data, the optimal mix parameters are identified as 15% coarse aggregate, 2% steel fibers, and 0.2% polypropylene fibers.

##### Phase II: Optimization of the Cementitious Material–Composite Fiber System

Phase II experiments focused on optimizing the synergistic effects between the supplementary cementitious material (fly ash) and the fiber reinforcement systems. As indicated in Table 9, the hierarchy of factors influencing mechanical properties varied slightly by metric: for compressive strength, the order of significance was fly ash > composite fiber > steel fiber; whereas for flexural strength, it was fly ash > steel fiber > composite fiber.

In terms of compressive strength, performance peaked at a fly ash content of 33%, with a modest 2.1% increase relative to 21%. However, exceeding the optimal point at 42% resulted in a significant 16.7% reduction in strength. Concurrently, increasing the steel fiber content to 1.5% and the composite fiber content to 0.5% enhanced compressive strength by 6.3% and 12.4%, respectively.

For flexural strength, a progressive decrease was observed with increasing fly ash content, with 21% identified as the optimal level. The incorporation of 1.5% steel fibers and 0.5% composite fibers significantly improved flexural performance by 16.1% and 14.0%, respectively. Based on these findings, the comprehensive optimal parameter range for Phase II is determined to be: fly ash 21–33%, steel fibers 1.5%, and composite fibers 0.5%.

#### 3.2.2. Analysis of Key Parameter Influence Mechanisms

The interplay between matrix packing density and fiber bridging efficiency governs the mechanical behavior of UHPC-CA. This section elucidates the distinct mechanisms of strengthening and weakening associated with the matrix constituents and reinforcement systems.

##### Mechanisms of Matrix Constituents

The contents of coarse aggregate and fly ash fundamentally alter the matrix microstructure. Coarse aggregate exerts a parabolic influence on strength; optimal dosages enhance particle packing and mechanical interlocking within the interfacial transition zone (ITZ), creating a rigid stress-transfer framework. However, excessive aggregate volumes compromise workability, disrupt fiber dispersion, and introduce internal defects that degrade performance. Similarly, fly ash exhibits a dual nature. Moderate substitution refines the pore structure via the physical micro-filling effect and secondary pozzolanic reactions. Conversely, excessive replacement diminishes the matrix’s intrinsic strength due to the limited reactivity and lower modulus of fly ash particles, causing a continuous decline in flexural strength and a threshold-dependent behavior in compressive strength.

##### Mechanisms of Fiber Reinforcement

The fiber reinforcement systems contribute through distinct multiscale mechanisms. Steel fibers, characterized by high tensile strength and modulus, provide the primary load-bearing capacity by effectively bridging macro-cracks and redistributing localized stresses. Consequently, both compressive and flexural strengths increase monotonically with steel fiber dosage. In contrast, polypropylene fibers primarily target early-stage micro-crack mitigation due to their high specific surface area. Their low modulus limits contributions to ultimate bearing capacity, and excessive dosages can induce porosity via air entrainment. Finally, composite fibers (polyethylene–polypropylene) offer a synergistic ductile mechanism. The high-modulus polyethylene component aids in stress transfer, while the hybrid system forms a multiscale crack-blocking network. This progressively enhances strength and ductility, provided that dosage levels are controlled to prevent porosity-induced strength regression.

#### 3.2.3. Comparative Analysis of Hybrid Fiber Systems

A comparative assessment reveals distinct performance trade-offs between the two fiber-reinforced systems. Substituting polypropylene and a portion of steel fibers with composite fibers results in a modest 12.0% reduction in peak compressive strength but yields a substantial 83.8% enhancement in flexural strength. These findings indicate that the composite fiber system effectively compensates for the reduced steel fiber content by forming a ductility, multiscale crack-bridging network that significantly amplifies ductility and deformability.

From a techno-economic perspective, while steel fibers offer superior load-bearing capacity, their widespread adoption is often constrained by cost. Conversely, polypropylene fibers are economical but provide limited structural reinforcement. The polyethylene–polypropylene composite system successfully bridges this gap, integrating the high-modulus benefits of synthetic fibers with cost efficiency. Consequently, partial replacement of steel fibers with composite fibers represents a rational optimization strategy. This approach synergistically balances mechanical performance and crack control while reducing material costs, offering a viable pathway for sustainable, high-performance, and ductile cementitious composite design.

### 3.3. Analysis of Variance (ANOVA)

**Figure 5:**
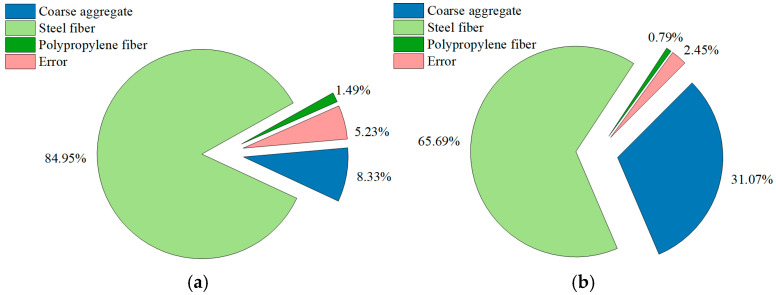
Contribution rate of each factor in Phase I experiments: (**a**) Compressive strength; (**b**) Flexural strength.

**Figure 6:**
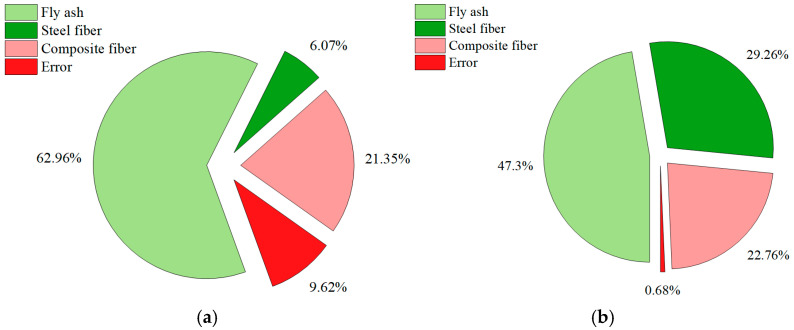
Contribution rate of each factor in Phase II experiments: (**a**) Compressive strength; (**b**) Flexural strength.

The results of the analysis of variance (ANOVA) for the Phase I and Phase II experiments are summarized in Table 10 and Table 11, respectively. Correspondingly, Figure 5 and Figure 6 depict the contribution ratios of each factor to the compressive and flexural strengths, respectively, as derived from these analyses. These results were used to determine the statistical significance of various reinforcement and admixture parameters on the mechanical performance of hybrid fiber-reinforced composites.

In Table 10 and Table 11, SS denotes the Sum of Squares, which reflects the contribution of each factor to the total variance. DOF represents the Degrees of Freedom, and MS denotes the Mean Square, corresponding to the average variance of a given factor. The MS value is compared with the Error term to determine statistical significance. The F-value indicates the test statistic, representing the ratio of the factor variance to the error variance. At the same time, Fa(2,2) refers to the critical F-value at the specified degrees of freedom. A factor is considered significant when its F-value exceeds the corresponding Fa(2,2) value. The significance symbols (*) and (*) * denote statistically substantial effects, respectively.

#### 3.3.1. Phase I Analysis

The Phase I ANOVA results (Table 10) indicate that the steel fiber had a statistically significant influence on both the compressive and flexural strengths of the specimens (F = 16.23 > Fa(2,2) = 0.05 and F = 26.80 > Fa(2,2) = 0.05, respectively). This finding demonstrates that steel fibers substantially enhance both the composite’s compressive and flexural strengths through their crack-bridging and stress-reduction effects. In contrast, the coarse aggregate exerted a significant effect only on flexural strength (F = 12.68 > Fa = 0.19), suggesting that the aggregate size and gradation contributed primarily to the matrix’s resistance against tensile cracking rather than compressive strength. The polypropylene fiber, with a much lower elastic modulus, showed no statistically significant improvement in either strength parameter, consistent with its role in crack control rather than strength development.

Overall, Phase I results revealed that steel fiber is the dominant factor influencing the composite’s mechanical response, particularly by improving both compressive and flexural strengths, whereas polypropylene fiber made a minor contribution.

#### 3.3.2. Phase II Analysis

The Phase II data (Table 11) indicate a shift in the dominant influencing factors due to the introduction of fly ash and composite fiber systems. The fly ash exhibited a significant effect on both compressive (F = 6.55 > Fa = 0.19) and flexural strengths (F = 69.68 > Fa = 0.19), highlighting its contribution to microstructural densification and improved matrix integrity through pozzolanic reactions. The steel fiber remained highly significant in flexural performance (F = 43.11 > Fa = 0.05) but lost relative significance in compressive strength, possibly due to the matrix modification induced by the incorporation of fly ash, which alters fiber–matrix interfacial bonding conditions.

Interestingly, the composite fiber system (combining steel and polypropylene fibers) demonstrated a marked synergistic effect on flexural behavior (F = 33.53 > Fa = 0.02). This outcome implies that the simultaneous presence of fibers with distinct mechanical characteristics led to a multiscale reinforcement mechanism in which steel fibers provided primary crack-bridging capacity. In contrast, polypropylene fibers delayed microcrack coalescence, collectively increasing energy absorption and post-cracking ductility.

#### 3.3.3. Comparative Interpretation

A comparison between Phase I and Phase II experiments suggests a transition from single-fiber-dominated strengthening to multi-component synergistic reinforcement. In Phase I, mechanical performance was primarily governed by discrete steel fiber reinforcement. In contrast, in Phase II, the combined action of fly ash and composite fibers established a more complex, interactive strengthening mechanism. The enhanced effect of fly ash on both strength indices indicates that matrix densification plays a more substantial role in the presence of hybrid fibers.

#### 3.3.4. Contribution Analysis Based on ANOVA Results

Figure 5 and Figure 6 present the contribution ratios of each factor to the mechanical performance of the composites in Phase I and Phase II, respectively, providing a graphical summary of the ANOVA results in Table 10 and Table 11.

In Phase I (Figure 5a), steel fiber accounts for 8.33% of the variance in compressive strength, the highest among the controllable factors. Coarse aggregate and polypropylene fiber contribute only 5.23% and 1.49%, respectively. In contrast, the error term accounts for 84.95%, indicating that compressive strength is primarily governed by intrinsic matrix properties and random variability, with steel fiber as the only effective strengthening parameter. For flexural strength (Figure 5b), the response is more factor-sensitive: steel fiber contributes 31.07%, coarse aggregate 2.45%, polypropylene fiber less than 1%, and the error decreases to 65.69%. Thus, flexural behavior is markedly more responsive to reinforcement composition, and steel fiber plays a particularly prominent role through crack-bridging and enhancement of post-cracking ductility, whereas polypropylene fibers exert negligible structural influence.

In Phase II (Figure 6a), the contribution pattern for compressive strength changes substantially. Fly ash dominates with a contribution of 62.96%, far exceeding that of the composite fiber system (21.35%) and steel fiber (6.07%), while the error term drops to 9.62%. This shift highlights the central role of fly ash in matrix densification via pozzolanic reactions and improved particle packing. For flexural strength (Figure 6b), contributions are more balanced: fly ash accounts for 47.30%, steel fiber 29.26%, and composite fiber 22.76%, with minimal error (0.68%). This distribution evidences a strong synergy between matrix modifiers (fly ash) and fiber reinforcements (steel and composite fibers), whereby matrix densification enhances load transfer and flexural stiffness, and the combined presence of steel and polypropylene fibers improves crack distribution uniformity and energy absorption.

Overall, Figure 5 and Figure 6 corroborate the ANOVA findings and clarify the quantitative roles of individual and combined factors. Steel fiber remains the primary contributor to flexural performance, fly ash emerges as the dominant factor for compressive strength through matrix densification, and composite fiber systems provide additional synergistic toughening under flexural loading. The integration of a densified matrix with multiscale fiber reinforcement thus yields statistically validated improvements in both strength and ductility, supporting the design of optimized hybrid fiber-reinforced composites.

### 3.4. Analysis of Fiber Interaction Effects Using Contour Maps

**Figure 7:**
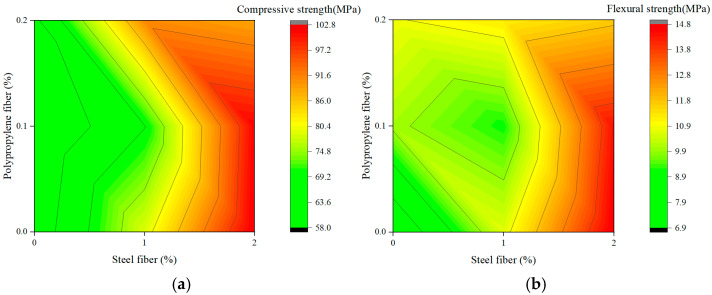
Contour maps of steel fiber—polypropylene fiber interaction: (**a**) Compressive strength; (**b**) Flexural strength.

**Figure 8:**
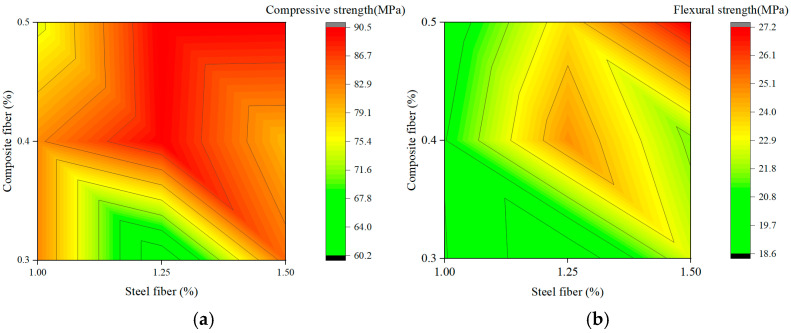
Contour maps of steel fiber–composite fiber interaction: (**a**) Compressive strength; (**b**) Flexural strength.

Figure 7 and Figure 8 present response-surface contour maps generated to visualize the coupling effects of fiber combinations. Mathematically, the topological features of these contours—specifically the gradient direction and curvature—serve as indicators of the sensitivity of the mechanical response to material variables and the magnitude of the interaction coefficients between them.

#### 3.4.1. Interaction Between Steel Fiber and Polypropylene Fiber

Figure 7 depicts the interaction between steel fiber (0–2%) and polypropylene fiber (0–0.2%) in Phase I. For compressive strength (Figure 7a), the contour lines are quasi-near and orthogonal to the steel fiber axis. This phenomenon indicates that the partial derivative of strength with respect to PP content approaches zero, implying that the regression coefficient for PP is statistically insignificant in this composite system. Compressive strength increases from approximately 58.0 MPa to over 102.8 MPa as steel fiber content rises to 2%, whereas changes in polypropylene fiber at a fixed steel level have minimal effect. This confirms that, within the investigated range, steel fibers are the primary contributors to compressive strength due to their high stiffness and crack-arresting capability. In contrast, low-modulus polypropylene fibers provide minor structural enhancement.

A similar but more pronounced trend is observed for flexural strength (Figure 7b). The response surface exhibits a steep gradient with increasing steel fiber content, with strength increasing from 6.9 MPa to 14.8 MPa. The contours are almost parallel to the *y*-axis, again highlighting the dominant influence of steel fibers through tensile stress transfer and crack bridging under flexure. The interaction with polypropylene fiber is weak, indicating no significant synergistic effect on flexural performance within the tested dosage window.

#### 3.4.2. Interaction Between Steel Fiber and Composite Fiber

Figure 8 shows the interaction between steel fiber (1.00–1.50%) and composite fiber (0.3–0.5%) in Phase II, where “composite fiber” denotes the polyethylene–polypropylene hybrid. For compressive strength (Figure 8a), the contour map reveals a clear two-factor interaction. The optimum region (>90.5 MPa) appears at simultaneously high levels of steel fiber (~1.50%) and composite fiber (~0.5%). The curved contour lines and gradients along both axes indicate that both variables significantly influence the response and that their effects are mutually dependent. This synergy is consistent with a multiscale reinforcement mechanism in which steel fibers control macro-cracks, while finer composite fibers stabilize micro-cracks and improve matrix homogeneity.

The interaction is even more evident for flexural strength (Figure 8b). The response surface exhibits a closed, elliptical high-value region, with the maximum strength (>27.2 MPa) attained at intermediate steel fiber (~1.25%) and composite fiber (~0.4%) contents. This elliptical contour pattern is characteristic of a strong positive interaction, implying that neither factor alone can achieve the peak response; instead, an optimal balance between the two is required. Mechanistically, steel fibers provide macroscopic toughness and ductility, whereas composite fibers enhance first-crack strength and energy absorption, thereby substantially improving flexural performance.

#### 3.4.3. Comparative Discussion

Comparison of Figure 7 and Figure 8 reveals a transition from a predominantly single-factor system to a genuinely synergistic hybrid system. In Phase I, mechanical behavior is essentially governed by steel fibers, with negligible interaction with polypropylene fibers. In Phase II, the combined and balanced presence of steel and composite fibers, together with the modified matrix, becomes critical for achieving optimal strength, particularly in flexure.

The contour maps thus provide clear visual evidence that a well-designed hybrid fiber system, exploiting multiscale reinforcement and positive interaction, outperforms simple single-fiber reinforcement. This supports the conclusion that optimizing the combination and balance of different reinforcement types is a more effective strategy for developing high-performance cementitious composites than merely increasing the dosage of a single fiber. This trend is consistent with the findings of Alwsabi et al. [14].

### 3.5. Microstructural Characterization and Analysis

#### 3.5.1. Microstructural Characterization of ITZ by SEM Observation

**Figure 9:**
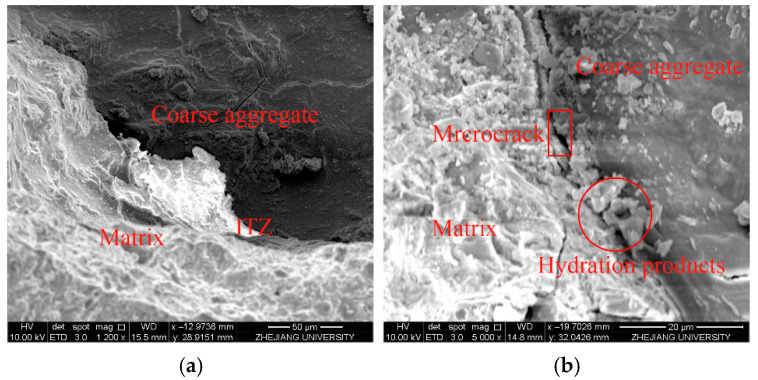

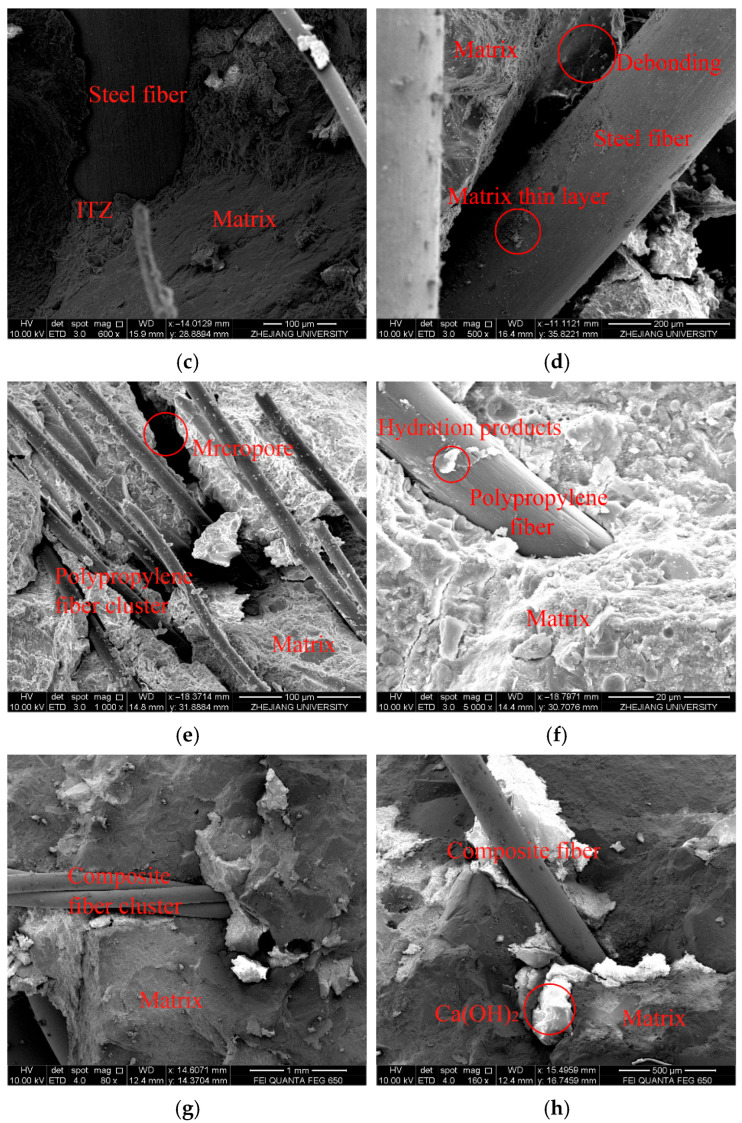
SEM micrographs of the interfacial transition zones (ITZ) between different components and the matrix: (**a**,**b**) coarse aggregate–matrix; (**c**,**d**) steel fiber–matrix; (**e**,**f**) polypropylene fiber–matrix; (**g**,**h**) composite fiber–matrix.

Figure 9 presents representative SEM images of the interfacial transition zones (ITZ) between aggregates, fibers, and the cementitious matrix, providing microstructural evidence for the macroscopic behaviors discussed above.

In the coarse aggregate–matrix region, a relatively porous ITZ appears as a light gray band with microcracks and isolated hydration products. Locally accumulated calcium hydroxide (CH) platelets and loosely packed hydrates (Figure 9b) are typical of wall-effect and bleeding-induced transition zones. These defects weaken interfacial bonding and are consistent with the limited strength contribution and low ANOVA contribution rates of coarse aggregate (Figure 5 and Figure 6).

By contrast, the steel fiber–matrix interface exhibits a compact, well-bonded ITZ (Figure 9c). Hydration products are densely concentrated around the fiber surface, forming strong mechanical interlock and enabling efficient stress transfer. Evidence of partial debonding with a residual matrix film adhering to the steel surface (Figure 9d) indicates a controlled pull-out mechanism rather than brittle interfacial failure. This damage mode accounts for the high energy absorption and enhanced flexural toughness observed for steel fibers in the mechanical and ANOVA results.

The polypropylene (PP) fiber–matrix interface (Figure 9e,f) shows a much weaker bond. Owing to the hydrophobic, smooth PP surface, voids and discontinuities are frequently observed along the interface, with irregular hydration residues on the fiber surface. This morphology is consistent with the negligible contribution of PP fibers to strength (Figure 5 and Figure 7). Nevertheless, thin hydration films and fine microcracks around PP fibers indicate localized stress redistribution and delayed crack initiation, supporting their role in microcrack control and durability enhancement rather than in load-bearing capacity.

In the hybrid system combining steel and polymer fibers, the ITZ becomes a denser, multiphase interfacial region. Fine Ca(OH)_2_ and C–S–H products are more uniformly distributed around both fiber types, with fewer microvoids and more continuous hydration layers than in single-fiber systems. This morphology suggests a cooperative reinforcement mechanism in which steel fibers carry the primary tensile load, while secondary fibers suppress microcrack propagation and stabilize the interface. The observed ITZ densification and multi-scale interlock (Figure 9g,h) are consistent with the contour-map results (Figure 8), which indicate that an optimized combination of steel and composite fibers produced peak compressive and flexural strengths.

Overall, the SEM observations establish a clear structure–performance correlation: the transition from weak, porous aggregate ITZs to dense, multi-phase fiber–matrix interfaces mirrors the shift from factor-dominated to synergistic reinforcement behavior. The hybrid fiber system effectively transforms the ITZ from a critical defect zone into an active strengthening region, underpinning the statistically verified gains in both strength and toughness.

#### 3.5.2. X-CT Analysis of Flexural Crack Development and Fiber Failure

**Figure 10:**
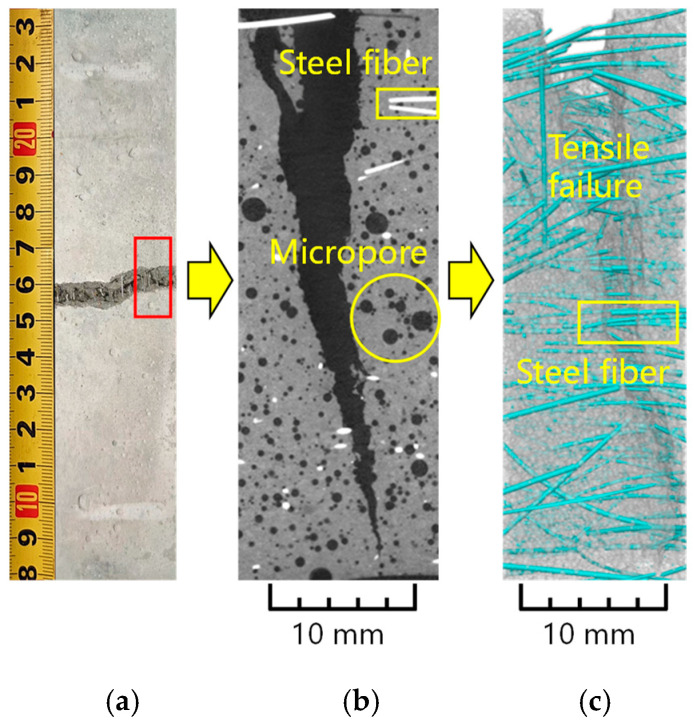
Flexural specimen X-CT diagram. (**a**) Failure specimen; (**b**) Development of main crack; (**c**) Distribution and failure of steel fiber.

Figure 10 illustrates the failure morphology and internal fiber distribution of the flexural specimen using X-ray computed tomography (X-CT).

The macroscopic surface view (Figure 10a) displays a typical flexural crack initiating from the tensile zone. The crack path is relatively tortuous rather than straight, and steel fibers are observed bridging the crack surfaces, indicating a non-brittle failure mode in which fibers actively resist crack opening.

The 2D X-CT cross-section (Figure 10b) reveals the internal crack propagation path. A primary crack extends through the matrix, bypassing more challenging phases. Notably, bright white spots represent the cross-sections of steel fibers, several of which are located directly across or near the crack path, confirming their role in bridging. The image also captures the distribution of internal micropores (dark circular voids), which appear randomly distributed within the matrix but do not seem to be the primary initiators of the main failure crack.

The 3D reconstruction (Figure 10c) provides a spatial visualization of the reinforcement network. The cyan cylinders represent steel fibers, which are generally well-dispersed throughout the specimen. In the tension failure zone (highlighted in the red box), multiple fibers clearly bridge the fracture plane. Some fibers appear pulled out or deformed, suggesting that the failure mechanism involves fiber–matrix debonding and pull-out, which contributes to energy dissipation and post-peak ductility.

The X-CT analysis confirms that the hybrid fiber system functions effectively under load. The randomized fiber network successfully intercepts crack propagation, transforming catastrophic rupture into a controlled, ductile failure process through fiber bridging and pull-out mechanisms.

### 3.6. Machine Learning-Based Strength Prediction

#### 3.6.1. Artificial Neural Network

**Figure 11:**
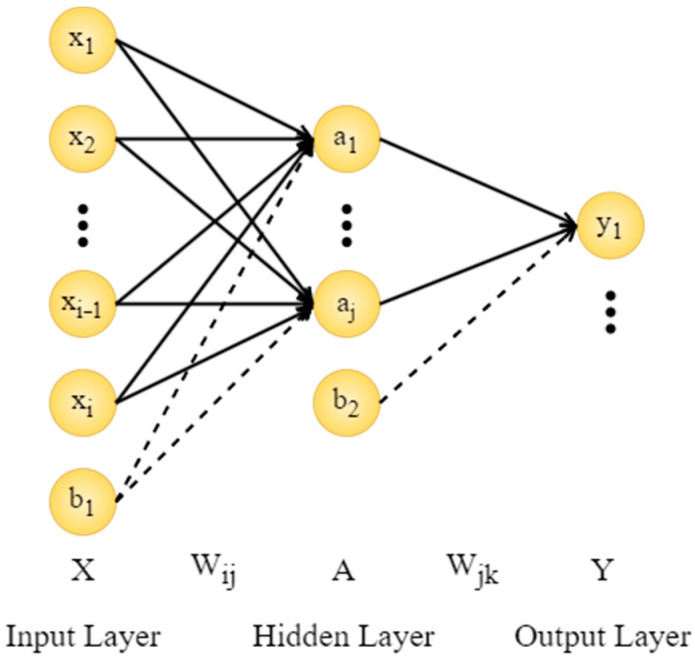
MLP network topology.

The artificial neural network (ANN) is a computational model inspired by biological neural systems and widely used to address complex nonlinear problems in engineering. Among various architectures, the multi-layer perceptron (MLP), a feed-forward network, is particularly effective in modeling nonlinear relationships between input factors and material properties. As illustrated in Figure 11, the MLP comprises an input layer, one or more hidden layers, and an output layer. Neurons within layers are connected by weighted links (W) with adjustable biases (b), which enhance model flexibility. Each neuron output is produced by applying a nonlinear activation function to the weighted sum of its inputs, enabling the network to capture the intricate interactions among compositional parameters governing compressive and flexural strengths.

The model is trained using the back-propagation (BP) algorithm, which optimizes weights and biases by minimizing prediction errors through iterative gradient-based learning. During this process, forward propagation computes model outputs, while backward propagation adjusts parameters to progressively reduce the loss function—typically measured by mean squared error (MSE).

Overall, the ANN provides a quantitative bridge between experimental data and predictive modeling. Its MLP structure forms the mathematical foundation for model training and validation, effectively representing the nonlinear, multi-factor synergy observed in the hybrid fiber system.

#### 3.6.2. Dataset and Evaluation Metrics

Based on the two-stage experimental program, a total of 50 data sets were collected. The output variable was compressive strength, ranging from 50.2 to 102.8 MPa, with a mean of 74.6 MPa. According to standard empirical guidelines, the number of input parameters should not exceed the square root of the dataset size. Accordingly, seven input variables were selected: water-to-binder ratio, fly ash content, coarse aggregate content, steel fiber content, polypropylene fiber content, composite fiber content, and curing age. The corresponding values for each variable are summarized in Table 12.

Model performance was evaluated using four statistical metrics: the root mean square error (RMSE), mean absolute error (MAE), coefficient of determination (R^2^), and the *a*20-index, as defined in Equations (2)–(5).

(2) $$RMSE=\sqrt{\frac{1}{n}\sum_{i=1}^{n}{\left(x_{i}-{\hat{x}}_{i}\right)}^{2}}$$

(3) $$MAE=\frac{1}{n}\sum_{i=1}^{n}\left|x_{i}-{\overset{⌢}{x}}_{i}\right|$$

(4) $$R^{2}=\frac{\sum_{i=1}^{n}{\left(x_{i}-\bar{x}\right)}^{2}-\sum_{i=1}^{n}{\left(x_{i}-{\bar{x}}_{i}\right)}^{2}}{\sum_{i=1}^{n}{\left(x_{i}-\bar{x}\right)}^{2}}$$

(5) $$a20-index=\frac{m20}{n}$$

where *n* is the number of samples; *x_i_* and ${\overset{⌢}{x}}_{i}$ denote the actual and predicted values of the *i*-th sample, respectively; $\bar{x}$ represents the mean of the actual values; and *m*20 is the number of samples whose ratio of actual to predicted values lies between 0.80 and 1.20. The *a*20-index, a recently proposed metric [34], is used to evaluate the MLP model’s prediction reliability.

#### 3.6.3. Training Process

The MLP model was trained using the PyTorch (version 2.2.1) deep learning framework. The training procedure consisted of five main steps:

(1) Data normalization. All input features were normalized to eliminate scale differences and improve convergence stability. The Z-score normalization method was adopted, as expressed in Equation (6):

(6) $$Y=\frac{X-\mu }{\sigma }$$

where *X* is the original input data, *µ* is the mean, and *σ* is the standard deviation of the dataset. This approach ensures that each input variable has a zero mean and unit variance, thereby enhancing the network’s training efficiency.

(2) Data partitioning. The entire dataset (50 samples) was randomly divided into two subsets: 80% for training and 20% for validation. The random sampling process was repeated five times to verify stability and reduce selection bias. Cross-validation [35] further ensured that the trained model had robust generalization capability and avoided overfitting.
(3) Network structure and optimization. Each MLP model comprised one input layer, one hidden layer, and one output layer. The input layer contained seven neurons corresponding to the seven experimental variables, while the output layer contained one neuron representing the predicted strength. The number of hidden neurons, denoted as m, was varied within the range [1, 10] to determine the optimal topology. The network optimization loss function, shown in Equation (7), was used to minimize prediction error during training [36]:

(7) $$L=\sqrt{m+n}+a$$

Although simplified here, this loss function follows the RMSE optimization principle, regulating weight updates via backpropagation to minimize the difference between actual and predicted outputs.

(4) Training and evaluation. The model employed the sigmoid activation function and used RMSE as the performance criterion. Learning rate was set to 0.1 based on empirical trials to ensure stable convergence. The training process was executed over multiple epochs. To ensure convergence and prevent overfitting, training was terminated after the model’s RMSE stabilized for more than 50 epochs. The stopping epoch was chosen based on the accuracy–cost trade-off observed during training (with an upper epoch cap), rather than an unjustified fixed rule. As shown in Table 13, multiple network structures were evaluated (e.g., 7-4-1, 7-8-1, 7-12-1), and their RMSE values were compared.
(5) Model optimization and selection. According to the training outcomes summarized in Table 13, the optimal network topology was 7-12-1, comprising seven input neurons, 12 hidden neurons, and one output neuron. This configuration achieved the lowest RMSE of 2.17, indicating high prediction accuracy and stable generalization.

#### 3.6.4. Performance Evaluation

**Figure 12:**
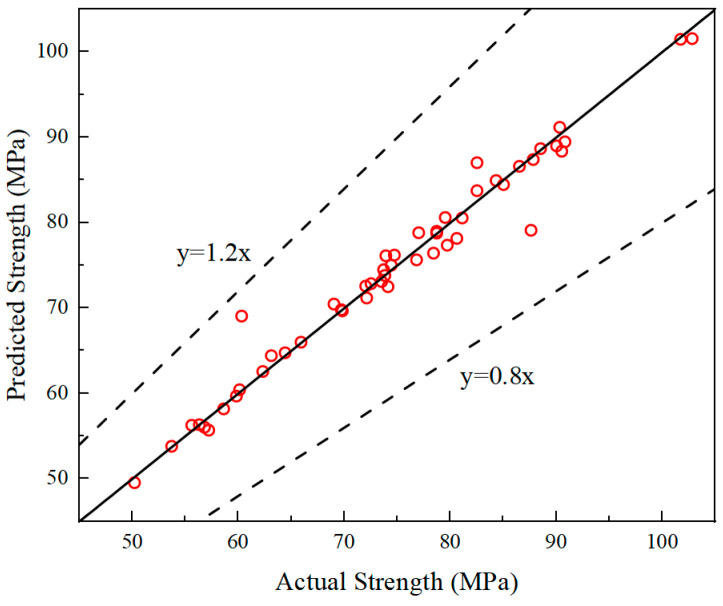
Regression plot between actual and predicted values.

The performance metrics of the proposed strength prediction model on the entire dataset are summarized in Table 14, and the correlation between predicted and actual values is shown in Figure 12. The model exhibits low RMSE and MAE, indicating minimal prediction error. Moreover, the high coefficient of determination (R^2^ = 0.9703) indicates excellent predictive performance. In addition, the *a*20-index reaches 1.0, indicating that 100% of the predicted values fall within the ±20% acceptable error range, confirming the model’s high accuracy. As shown in Figure 12, most data points are concentrated near the ideal regression line (y = x), with only a few outliers. This close alignment between predicted and measured strength values demonstrates the model’s superior linear fitting, strong prediction stability, and overall reliability. Therefore, the developed strength prediction model provides highly accurate estimations and exhibits strong potential for practical engineering applications.

**Figure 13:**
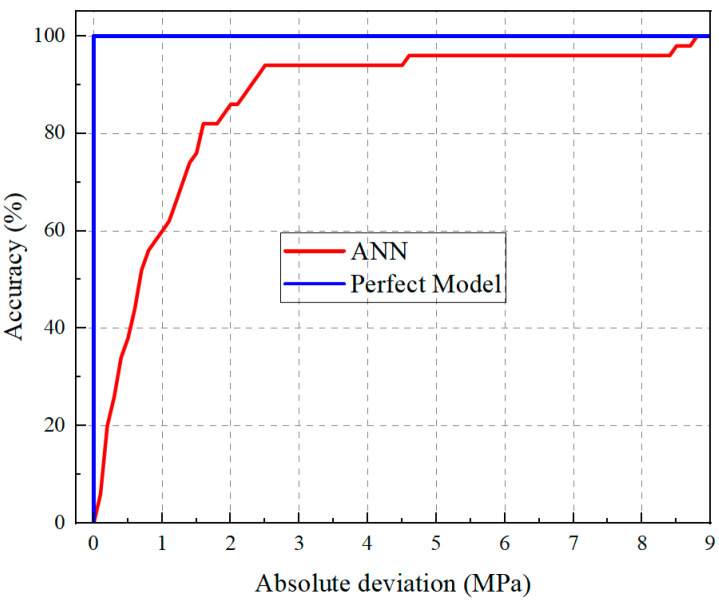
REC curve.

To comprehensively assess the predictive capability of the proposed model, the Regression Error Characteristic (REC) curve was constructed from the absolute deviations between predicted and measured values, as illustrated in Figure 13. In this figure, the horizontal axis represents the error tolerance, and the vertical axis denotes the cumulative proportion of predictions that fall within the specified error range. A model exhibiting a minor overall absolute error—corresponding to a larger area under the REC curve—is indicative of superior predictive performance [37]. As shown in Figure 13, the REC curve of the proposed model closely follows the ideal reference line, demonstrating excellent fitting accuracy and generalization. Furthermore, most prediction errors fall well within the acceptable precision threshold, confirming the robustness and reliability of the developed model.

#### 3.6.5. Model Interpretability and Feature Importance Analysis Based on SHAP Values

**Figure 14:**
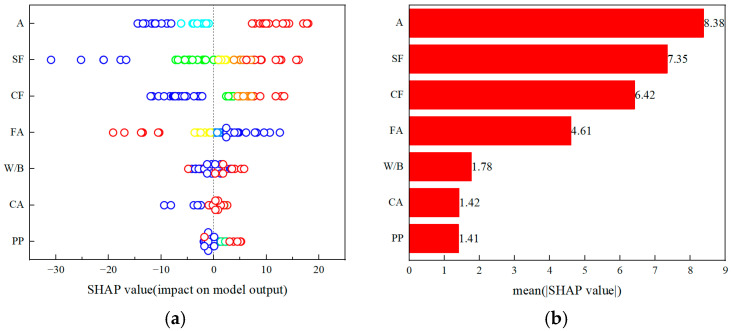
SHAP-based global interpretation of input features: (**a**) SHAP value distribution indicating variable contribution and direction of influence; (**b**) Mean absolute SHAP values showing the relative importance of each variable.

To elucidate the influence of each input variable on the model output, SHAP (Shapley Additive Explanations) analysis was conducted to provide a global interpretation of feature contributions [38], as illustrated in Figure 14. In Figure 14a, the vertical axis lists the input variables, while the horizontal axis represents their corresponding SHAP values. A positive SHAP value indicates that the variable contributes to an increase in the predicted compressive strength, whereas a negative value suggests the opposite. The color gradient from blue to red denotes the relative magnitude of each variable, with blue representing lower input values and red representing higher ones. In Figure 14b, the horizontal axis shows the mean absolute SHAP value of each feature, quantifying its average contribution to the model prediction.

As seen in Figure 14a, all variables—except fly ash content (FA)—exhibit a positive correlation with compressive strength. Variations in age (A), steel fiber content (SF), composite fiber content (CF), and fly ash content (FA) substantially affect predicted strength, indicating that the model output is susceptible to these parameters. Conversely, the water-to-binder ratio (W/B), coarse aggregate content (CA), and polypropylene fiber content (PP) exert a comparatively minor influence. Correspondingly, Figure 14b shows that the most influential features governing compressive strength are age, steel fiber content, composite fiber content, and fly ash content, confirming their dominant role in the model’s strength prediction mechanism.

## 4. Conclusions

This study proposes an integrated “experiment–characterization–modeling” framework to evaluate the mechanical performance of UHPC-CA. Based on the orthogonal experimental results, microstructural analysis, and ANN modeling, the following conclusions are drawn:

(1) Synergistic Reinforcement and Quantitative Enhancement: The hybrid system combining steel and composite fibers demonstrated superior performance compared to the traditional steel–polypropylene combination. Specifically, the hybrid group achieved a 12.4% increase in compressive strength and a 14% improvement in flexural strength. This suggests that the partial substitution of steel fibers with composite fibers is a viable strategy for balancing cost and ductility. However, the optimal substitution ratio is sensitive to the total fiber volume.
(2) Coupled Optimization of Fly Ash and Fibers: Fly ash functions beyond a mere filler; its optimal dosage (21–33%) densifies the matrix and refines the fiber–matrix interface. The study demonstrates that mineral admixtures and fiber systems exhibit strong coupled effects on strength development, necessitating a co-optimization approach rather than independent parameter selection to maximize packing density and bonding efficiency.
(3) Microstructural Mechanisms of Ductility: SEM and X-CT analyses provide physical evidence connecting macroscopic performance to micromorphology. The enhancement in ductility is attributed to a densified ITZ and a multi-scale crack-bridging network. The hybrid fibers were observed to alter the failure mode from brittle fracture to controlled ductile rupture, physically supporting the multi-crack propagation behavior observed in the mechanical tests.
(4) Data-Driven Prediction via ANN: The developed ANN model effectively captured the non-linear interactions among mix parameters, achieving a high accuracy with an R2 of 0.9703 and an RMSE of 2.11 MPa for compressive strength. The sensitivity analysis further identified that fiber volume and fly ash content are the most critical variables affecting the mechanical output within the tested domain.
(5) Limitations and Future Perspectives: While the proposed framework shows promise, it is essential to note the limitations regarding the sample size and data distribution. The current ANN model provides reliable predictions within the specific parameter range of this study; however, extrapolation to mix designs with significantly different aggregate types or fiber geometries may introduce errors and require caution. Therefore, future work should focus on expanding the dataset to improve model generalization. Additionally, to complement the current microstructural analysis, future studies should incorporate in situ micromechanical characterization (e.g., fiber pull-out tests within an SEM or nanoindentation) to provide a more dynamic understanding of interface behavior. Finally, investigating the long-term durability (e.g., chloride resistance) of the UHPC-CA hybrid fiber system is also recommended for practical applications.

## Figures and Tables

**Table 1:** Chemical composition and content of cement (%).

Insoluble Matter	Loss on Ignition	MgO	S_2_O_3_	Chloride Ion	Limestone	Natural Gypsum
0.95	2.13	1.46	2.59	0.019	2.16	6.54

**Table 2:** Chemical composition and content of fly ash (%).

Moisture Content	Loss on Ignition	Water Requirement Ratio	S_2_O_3_	Free CaO	Total Mass Fraction of SiO_2_, Al_2_O_3_, and Fe_2_O_3_	28 d Activity Index
0.02	1.80	93	0.7	0.05	77	79

**Table 3:** Chemical components and content of fine sand (%).

SiO_2_	Fe_2_O_3_	Al_2_O_3_	TiO_2_	CaO	MgO	Na_2_O	K_2_O
93.0	0.31	4.63	0.057	0.23	0.12	1.38	2.18

**Table 4:** Physical property parameters of different fibers.

Fiber Types	Tensile Strength (MPa)	Length (mm)	Equivalent Diameter (mm)	Initial Modulus (GPa)
Steel	2850	13.0	0.220	200
Polypropylene	>350	19.0	0.034	>3.5
Polyethylene–polypropylene	632	12.95	0.021	13.2

**Table 5:** The benchmark mix ratio (kg/m^3^).

Cement	Fly Ash	Silica Fume	Sand	Coarse Aggregate	Steel Fiber	Plasticizer	Water
600	100	50	1000	450	156	5	250

**Table 6:** Experimental Parameters and Test Results of Phase I.

Specimen	Coarse Aggregate (%)	Steel Fiber (%)	Polypropylene Fiber (%)	Compressive Strength (MPa)	Flexural Strength (MPa)
A-1	10	0	0	60.1	6.9
A-2	10	1	0.1	69.0	9.1
A-3	10	2	0.2	88.5	11.7
A-4	15	0	0.1	58.0	10.0
A-5	15	1	0.2	90.8	11.2
A-6	15	2	0	102.8	14.8
A-7	20	0	0.2	62.3	10.8
A-8	20	1	0	78.7	10.6
A-9	20	2	0.1	101.7	14.4

**Table 7:** Experimental Parameters and Test Results of Phase II.

Specimen	Fly Ash (%)	Steel Fiber (%)	Composite Fiber (%)	Compressive Strength (MPa)	Flexural Strength (MPa)
B-1	21	1	0.3	82.5	20.8
B-2	21	1.25	0.4	90.0	24.8
B-3	21	1.5	0.5	90.3	27.2
B-4	33	1	0.4	82.5	20.7
B-5	33	1.25	0.5	90.5	23.5
B-6	33	1.5	0.3	84.3	22.6
B-7	42	1	0.5	74.4	20.0
B-8	42	1.25	0.3	60.3	18.6
B-9	42	1.5	0.4	79.7	21.6

**Table 8:** Range Analysis of Phase I Experiments.

Index	Level	Coarse Aggregate	Steel Fiber	Polypropylene Fiber
Compressive strength (MPa)	K1	72.5	60.1	80.5
	K2	83.9	79.5	76.2
	K3	80.9	97.7	80.5
	R	11.4	37.6	4.3
Flexural strength (MPa)	K1	9.2	9.2	10.8
	K2	12.0	10.3	11.2
	K3	11.9	13.6	11.2
	R	2.8	4.4	0.4

**Table 9:** Range Analysis of Phase II Experiments.

Index	Level	Fly Ash	Steel Fiber	Composite Fiber
Compressive strength (MPa)	K1	85.8	79.8	75.7
	K2	87.6	80.3	84.1
	K3	71.5	84.8	85.1
	R	16.1	5.0	9.4
Flexural strength (MPa)	K1	24.3	20.5	20.7
	K2	22.3	22.3	22.4
	K3	20.1	23.8	23.6
	R	4.2	3.3	2.9

**Table 10:** ANOVA Results of the Phase I Experiment.

Index	Factor	SS	DOF	MS	F	Fa(2,2)	Significance
Compressive strength	Coarse aggregate	207.25	2	103.63	1.59	0.19	
	Steel fiber	2113.85	2	1056.93	16.23	0.05	(*)
	Polypropylene fiber	36.98	2	18.49	0.28	0.02	
	Error	130.24	2	65.12			
Flexural strength	Coarse aggregate	14.95	2	7.48	12.68	0.19	(*)
	Steel fiber	31.61	2	15.81	26.80	0.05	(*) *
	Polypropylene fiber	0.38	2	0.19	0.32	0.02	
	Error	1.18	2	0.59			

**Table 11:** ANOVA Results of the Phase II Experiment.

Index	Factor	SS	DOF	MS	F	Fa(2,2)	Significance
Compressive strength	Fly ash	468.14	2	234.07	6.55	0.19	
	Steel fiber	45.14	2	22.57	0.63	0.05	
	Composite fiber	158.74	2	79.37	2.22	0.02	
	Error	71.49	2	35.75			
Flexural strength	Fly ash	26.48	2	13.24	69.68	0.19	(*) *
	Steel fiber	16.38	2	8.19	43.11	0.05	(*) *
	Composite fiber	12.74	2	6.37	33.53	0.02	(*) *
	Error	0.38	2	0.19			

**Table 12:** The values of input variables.

Parameter	Level 1	Level 2	Level 3	Level 4	Level 5
Water-binder ratio	0.33	0.27	/	/	/
Fly ash (%)	17	21	33	42	/
Coarse aggregate (%)	10	15	20	/	/
Steel fiber (%)	0	1	1.25	1.5	2
Polypropylene fiber (%)	0	0.1	0.2	/	/
Composite fiber (%)	0.3	0.4	0.5	/	/
Age (d)	7	14	28	/	/

**Table 13:** Training results.

Network Topology	RMSE
7-4-1	6.73
7-5-1	4.19
7-6-1	4.68
7-7-1	4.63
7-8-1	2.83
7-9-1	2.60
7-10-1	3.02
7-11-1	2.84
7-12-1	2.17

**Table 14:** Details of performance indices.

Network Topology	RMSE	MAE	R^2^	*a*20-Index
7-12-1	2.11	1.22	0.9703	1

## Data Availability

The original contributions presented in this study are included in the article. Further inquiries can be directed to the corresponding author.
